# Retinoic Acid and POU Genes in Developing Amphioxus: A Focus on Neural Development

**DOI:** 10.3390/cells12040614

**Published:** 2023-02-14

**Authors:** Matteo Bozzo, Deianira Bellitto, Andrea Amaroli, Sara Ferrando, Michael Schubert, Simona Candiani

**Affiliations:** 1 Dipartimento di Scienze della Terra dell’Ambiente e della Vita (DISTAV), Università degli Studi di Genova, 16132 Genoa, Italy; 2 Laboratoire de Biologie du Développement de Villefranche-sur-Mer (LBDV), Institut de la Mer de Villefranche, Sorbonne Université, CNRS, 06230 Villefranche-sur-Mer, France

**Keywords:** all-*trans* retinoic acid, BMS493, cephalochordates, evolution of development, GABAergic neurons, GAD, HCR, neural patterning, *Pou4*, terminal selector

## Abstract

POU genes are a family of evolutionarily conserved transcription factors with key functions in cell type specification and neurogenesis. In vitro experiments have indicated that the expression of some POU genes is controlled by the intercellular signaling molecule retinoic acid (RA). In this work, we aimed to characterize the roles of RA signaling in the regulation of POU genes in vivo. To do so, we studied POU genes during the development of the cephalochordate amphioxus, an animal model crucial for understanding the evolutionary origins of vertebrates. The expression patterns of amphioxus POU genes were assessed at different developmental stages by chromogenic in situ hybridization and hybridization chain reaction. Expression was further assessed in embryos subjected to pharmacological manipulation of endogenous RA signaling activity. In addition to a detailed description of the effects of these treatments on amphioxus POU gene expression, our survey included the first description of *Pou2* and *Pou6* expression in amphioxus embryos. We found that *Pit-1*, *Pou2*, *Pou3l*, and *Pou6* expression are not affected by alterations of endogenous RA signaling levels. In contrast, our experiments indicated that *Brn1/2/4* and *Pou4* expression are regulated by RA signaling in the endoderm and the nerve cord, respectively. The effects of the treatments on *Pou4* expression in the nerve cord revealed that, in developing amphioxus, RA signaling plays a dual role by (1) providing anteroposterior patterning information to neural cells and (2) specifying neural cell types. This finding is coherent with a terminal selector function of *Pou4* for GABAergic neurons in amphioxus and represents the first description of RA-induced changes in POU gene expression in vivo.

## 1. Introduction

POU genes are a group of homeobox genes encoding DNA-binding transcription factors. The evolutionary origins of POU genes likely date back to the origin of metazoan animals, with representatives having been identified in all major metazoan groups, including sponges [1]. Despite this ancient origin, POU genes have conserved functions in cell type specification and neurogenesis across metazoans [2]. The POU gene family can be subdivided into six classes (I–VI), named *Pou1*–*Pou6*, with suffixes (*f1–f4*) being used to discriminate between paralogs when more than one family member is present in a given class [3]. However, the historical, pre-nomenclature names are still widely used for some POU genes. We will thus use these historical gene names in this work but provide a table cross-referencing the different names given to POU genes to avoid confusion (Table 1).

Retinoic acid (RA) is an important morphogen during vertebrate development [4]. RA signaling mostly acts by binding to heterodimers of two nuclear hormone receptors, the retinoic acid receptor (RAR) and the retinoid X receptor (RXR), which act as ligand-activated transcription factors and thus promote transcription of target genes in the presence of RA [5]. RA signaling evolved early in bilaterians, likely as a low-affinity sensor system. High-affinity RAR-mediated RA signaling seems to be more recent and specific to the chordate lineage [6,7]. The origin of chordates therefore marked an important turning point in the evolution of RA signaling.

Amphioxus (subphylum Cephalochordata) represents the earliest branching chordate group and therefore the sister group of all other chordates, namely tunicates and vertebrates [8]. Since amphioxus evolves slowly and has a prototypical chordate body plan (reviewed in [9]), it is a key model for understanding chordate diversification and vertebrate evolution. The developing nerve cord of amphioxus, for example, presents homologies to the main regions of the vertebrate central nervous system (CNS) [10,11,12], containing well-characterized neurotransmitter systems [13,14,15,16] and glial cells similar to vertebrate astroglia and radial glia [17]. Amphioxus possesses at least one member of each POU class except class V, which is limited to vertebrates (Table 1) [18]. Regarding RA signaling, amphioxus has an RA signaling system similar to that of vertebrates, with a high-affinity RAR regulating anteroposterior patterning of the CNS by directly controlling the colinear expression of *Hox* genes [19,20]. In addition, RA signaling is involved in patterning the endoderm, general ectoderm, and peripheral nervous system (PNS) [21,22,23,24,25].

**Table 1:** POU family members in vertebrates and cephalochordates.

Class	Vertebrates	Cephalochordates
I	*Pou1f1* (*Pit1*; *Ghf1*)	*Pit-1 (AmphiPOU1F1)* [26,27,28]
II	*Pou2f1* (*Oct1*; *Otf1*)	
	*Pou2f2* (*Oct2*; *Otf2*)	*Pou2* [18]
	*Pou2f3* (*Oct11*; *Skn1a*; *Pla1*; *Epoc1*)	
III	*Pou3f1* (*Oct6*; *Otf6*; *Scip; Tst1*; *Xlpou1*)	
	*Pou3f2* (*Oct7*; *Otf7; Brn2*; *Xlpou3*)	*Brn1/2/4 (Pou3f)* [29,30]
	*Pou3f3* (*Oct8*; *Otf8*; *Brn1*)	*Pou3l (Pou3fl)* [18,30]
	*Pou3f4* (*Oct9*; *Otf9*; *Brn4*; *Dfn3*; *Xlpou2*)	
IV	*Pou4f1 (Brn3a; Rdc-1; Oct-T1)*	
	*Pou4f2 (Brn3b; Brn-3.2)*	*Pou4* (*AmphiPOU-IV*) [31]
	*Pou4f3 (Brn3c; Brn-3.1; Dfna15)*	
V	*Pou5f1* (*Oct3*; *Oct4*; *Otf3*; *Otf4*)	None [18]
	*Pou5f2* (*Sprm1; Pou2/V; Pou5f3*)	
VI	*Pou6f1 (Brn5; Mpou*; *Tcfb1)Pou6f2 (Emb; Rpf1)*	*Pou6* [18]

In vitro evidence suggests that RA signaling controls cell-type-specific differentiation processes by regulating the expression of different POU transcription factors [32,33]. For example, *Pou3f2* (*Brn2*) is upstream of a gene regulatory network that, in response to RA treatment, directs embryonic stem cell differentiation towards a glutamatergic neuron fate [34]. Moreover, it has been suggested that RA signaling regulates the differential expression of two *Pou4* paralogs in the neural tube of developing mice [32]. However, studies correlating the functions of RA signaling and POU genes in vivo are still lacking. Therefore, with this work, we aimed to investigate the roles of RA signaling in the regulation of POU gene expression during amphioxus development. We thus performed pharmacological treatments to alter RA signaling activity during amphioxus development and analyzed the effects of the treatments on the expression of amphioxus POU genes. Our results provide the first evidence that RA signaling can act through a POU gene in vivo and suggest that *Pou4* has a conserved role as a terminal selector for GABAergic cell types in metazoans. Taken together, our data are consistent with the notion that, in the developing amphioxus nerve cord, RA signaling provides anteroposterior patterning information to neural cells and specifies neural cell types.

## 2. Materials and Methods

### 2.1. Spawning, Embryo Collection, and Pharmacological Treatments

Sexually mature adults of the European amphioxus (*Branchiostoma lanceolatum*) were collected in Argelès-sur-Mer, France, [35] and maintained in a seawater facility until the spawning season. Amphioxus embryos were obtained by in vitro fertilization using gametes released in response to a thermal shock as previously described [36]. To interfere with endogenous RA signaling, embryos were continuously treated from the G1 stage (6 hpf, hours post fertilization) to the T1 stage (36 hpf) with either 0.1 μM all-*trans* retinoic acid RA or 1 μM BMS493, both purchased from Sigma-Aldrich (Saint-Quentin Fallavier, France). RA is the endogenous ligand of RAR and therefore activates RA signaling. Conversely, BMS493 is a pan-RAR inverse agonist which stabilizes the binding of nuclear receptor corepressor proteins to the ligand-binding domain of RAR, thus hampering the binding of RA to RAR [37]. BMS493 therefore acts as an antagonist of RA signaling. Control embryos were treated with 0.1% dimethyl sulfoxide (DMSO), the solvent used to dissolve both RA and BMS493. A detailed protocol has been presented elsewhere [36]. Embryos were raised at 19 °C and staged according to [38].

### 2.2. Gene Cloning and RNA Probe Synthesis

*B. lanceolatum Pou* genes were identified by BLAT searches on genomic and transcriptomic resources [39] available on the UCSC Genome Browser (https://ucsc.crg.eu/, last accessed on 9 August 2022) using the sequences of the *B. floridae Pou* genes as queries [18]. Total RNA was extracted from embryos at different developmental stages using TRIzol (Thermo Fisher Scientific, Waltham, MA, USA), and cDNA was synthesized using the SuperScript III Reverse Transcriptase Kit (Thermo Fisher Scientific, Waltham, MA, USA). Partial sequences of *B. lanceolatum* POU genes were amplified by RT-PCR using the primers listed in Table S1. Amplicons isolated by PCR were cloned into the pCRII-TOPO vector (Life Technologies, Carlsbad, CA, USA) and used to synthesize DIG-labeled sense and antisense riboprobes using the DIG RNA Labeling Kit (Roche, Basel, Switzerland), following the manufacturer’s instructions. Results of control in situ hybridization experiments with sense probes are presented in Figure S1.

### 2.3. Embryo Fixation, Whole Mount In Situ Hybridization, Hybridization Chain Reaction, and Imaging

Embryos and larvae were fixed in 4% paraformaldehyde in 3-(N-morpholino)propanesulfonic acid buffer (see [36] for details) and stored in 70% ethanol. Chromogenic whole mount in situ hybridization was performed according to a published protocol [36]. Stained specimens were mounted in 90% glycerol and photographed using an IX71 inverted microscope (Olympus, Hamburg, Germany) equipped with a ColorViewII camera (Olympus, Hamburg, Germany).

Co-localization of *Pou4* and *GAD* was assessed by in situ hybridization chain reaction (HCR) v3.0 [40]. To design probes, the open reading frames of the genes of interest were submitted to the in situ probe generator from the Özpolat laboratory [41] (https://github.com/rwnull/insitu_probe_generator, last accessed on 9 August 2022). The sequences obtained were used to order DNA oligo pools from Integrated DNA Technology (Coralville, IA, USA), which were resuspended to a concentration of 1 μM in RNase-free water. The following HCR amplifiers with fluorophores were purchased from Molecular Instruments (Los Angeles, CA, USA): B1-H1-546, lot: S049723; B1-H2-546, lot: S019624; B2-H1-647, lot: S013724; B2-H2-647, lot: S017524. Double in situ HCR was performed according to the protocol from the Patel laboratory [42]. For both genes, the expression patterns obtained were identical to those observed using chromogenic in situ hybridization (this paper and [21]). Embryos were counterstained with Hoechst, mounted in 90% glycerol, and imaged with a TCS SP8 CLS microscope (Leica, Wetzlar, Germany) at 20x magnification. Brightness–contrast adjustments and z-projections were performed in Fiji [43]. Single images were assembled into figures using Photoshop CS4 (Adobe, San Jose, CA, USA).

### 2.4. Statistical Analyses

To evaluate the effects of RA signaling manipulation on the number of *Pou4*-positive cells in the nerve cord, T1-stage embryos were photographed in dorsal views following in situ hybridization as described in Section 2.3. For each specimen, the number of *Pou4*-positive cells anterior and posterior to the first pigment spot was counted. To confirm that the differences observed between treated (BMS493, *n* = 12; RA, *n* = 11) and control (DMSO, *n* = 13) embryos were statistically significant, the following analyses were performed. (1) The presence of outliers was tested with the ROUT method. (2) Data distribution was evaluated using a normality and lognormality test (Kolmogorov–Smirnov). (3) Since the data showed non-normal distribution, a multiple comparison analysis was performed with a one-way ANOVA Kruskal–Wallis test (Dunn’s multiple comparisons test). All *p*-values < 0.01 were considered statistically significant. Statistical analyses were carried out using Prism 8.0.1 (GraphPad Software, San Diego, CA, USA).

## 3. Results

Our analyses retrieved six POU genes in the *B. lanceolatum* genome, namely *Pit-1*, *Pou2*, *Pou3l*, *Brn1/2/4*, *Pou4*, and *Pou6*, confirming a previous report on *B. floridae* POU genes [18]. The amphioxus genome hence encodes single orthologs of POU classes I, II, IV, and VI (*Pit-1*, *Pou2*, *Pou4*, and *Pou6*, respectively), no ortholog of POU class V, and two orthologs of POU class III (*Brn1/2/4* and *Pou3l*). Of the two amphioxus POU class III genes, the sequence of *Brn1/2/4* is much more similar to the POU3 genes of vertebrates, with that of *Pou3l* having diverged significantly [18]. The expression patterns during normal development have already been described for some of the amphioxus POU genes: for *Pit-1* [26], *Brn1/2/4* [29], and *Pou4* [31] in *B. floridae* and for *Brn1/2/4* and *Pou3l* in *B. lanceolatum* [30]. The expression patterns of amphioxus *Pou2* and *Pou6* are thus described here for the first time.

### 3.1. Retinoic Acid Signaling Does Not Affect Pit-1, Pou2, Pou3l, and Pou6 Expression in Developing Amphioxus

**Figure 1:**
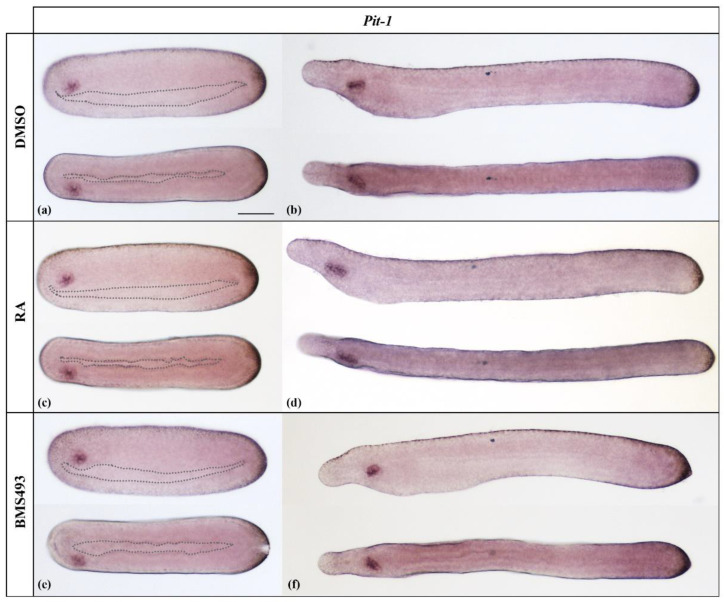
Expression of *Pit-1* in N4- and T1-stage *Branchiostoma lanceolatum* embryos. (**a**,**b**) Control embryos (DMSO-treated). (**c**,**d**) Retinoic acid (RA) signaling activation with all-*trans* retinoic acid (RA). (**e**,**f**) RA signaling inhibition with the inverse agonist BMS493. *Pit-1* is expressed exclusively in the preoral organ, and this expression is unaffected by the treatments. Whole mount embryos are shown in lateral (top) and dorsal (bottom) views; the anterior is to the left. Dotted outlines mark the borders of the gut in N4 neurulae. The scale bar in (**a**) is 50 μm and is valid for all panels.

In line with the expression pattern described for *B. floridae* [26], *Pit-1* started to be expressed in N4 neurulae only in Hatschek’s left diverticulum, an evagination of the left wall of the anterior archenteron (Figure 1a). In T1-stage embryos, Hatschek’s left diverticulum completed its separation from the endoderm and formed the preoral organ, which maintained the expression of *Pit-1* (Figure 1b). This expression pattern was not altered by ectopic activation (Figure 1c,d) or inhibition (Figure 1e,f) of RA signaling.

**Figure 2:**
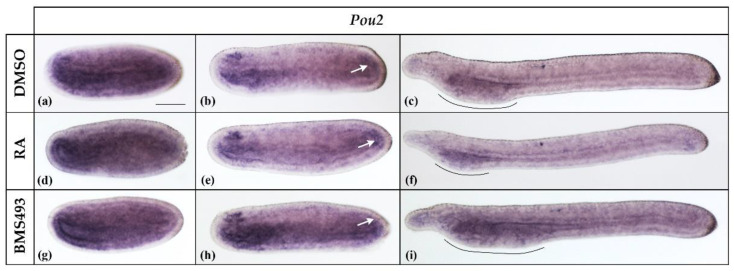
Expression of *Pou2* in N2-, N4-, and T1-stage *Branchiostoma lanceolatum* embryos. (**a**–**c**) Control embryos (DMSO-treated). (**d**–**f**) Retinoic acid (RA) signaling activation with all-*trans* retinoic acid (RA). (**g**–**i**) RA signaling inhibition with the inverse agonist BMS493. *Pou2* expression is widespread in the embryo and slightly more conspicuous in the pharyngeal endoderm and anterior neural plate in N2 neurulae (**a**,**d**,**g**). At the N4 stage, expression is restricted to the endoderm, the anterior nerve cord, and the tail bud region (white arrows) (**b**,**e**,**h**). *Pou2* expression is subsequently downregulated and becomes restricted, at the T1 stage, to the pharyngeal region, with the extent of the *Pou2* signal depending on the overall size of the pharynx (pharynx extent delimited by black lines) (**c**,**f**,**i**). Whole mount embryos are shown in lateral views; the anterior is to the left. The scale bar in (**a**) is 50 μm and is valid for all panels.

*Pou2* transcripts were already expressed at stage N2, the earliest we analyzed. At this stage, *Pou2* was expressed diffusely in the embryo and slightly more conspicuously in the pharyngeal endoderm and the anterior neural plate (Figure 2a). At the N4 stage, the expression became restricted to the endoderm, the neural plate, and the tail bud region (Figure 2b, white arrow), with the strongest signal being detectable in the anterior neural plate in a region corresponding to the prospective cerebral vesicle. By the stage T1, *Pou2* was downregulated in the nervous system and became restricted to the pharyngeal endoderm (Figure 2c). Although the treatments with RA and BMS493 did not directly affect the *Pou2* expression pattern, the *Pou2* expression domain in the pharyngeal endoderm decreased and increased in size according to the respective effects of the treatments on the overall length of the pharynx, with RA decreasing and BMS493 increasing it [23,44,45].

**Figure 3:**
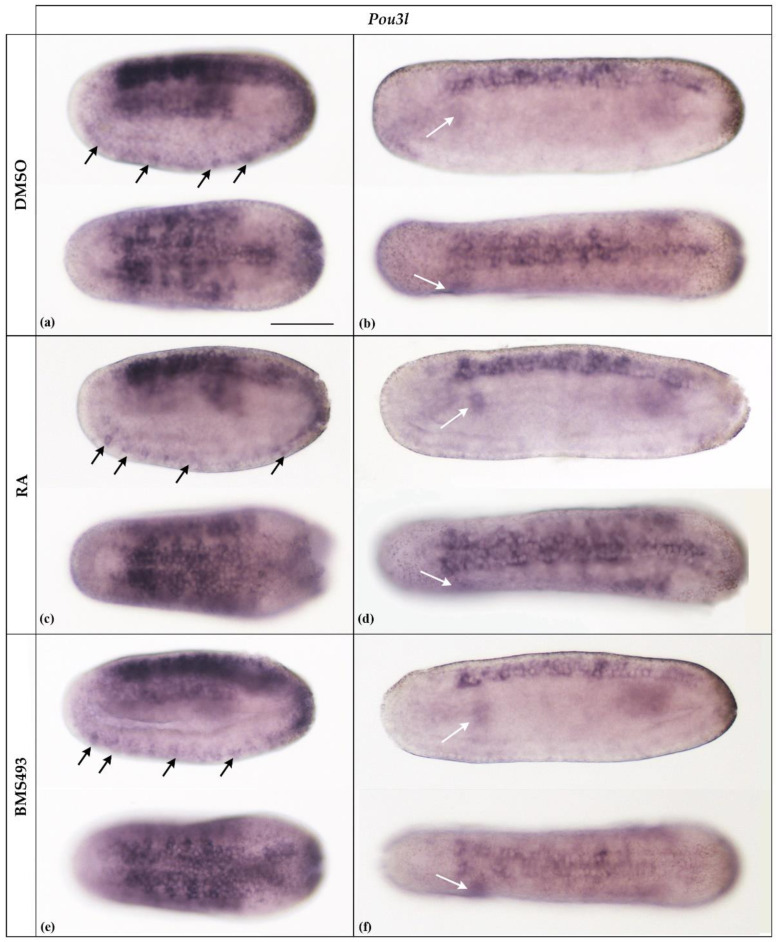
Expression of *Pou3l* in N2- and N4-stage *Branchiostoma lanceolatum* embryos. (**a**,**b**) Control embryos (DMSO-treated). (**c**,**d**) Retinoic acid (RA) signaling activation with all-*trans* retinoic acid (RA). (**e**,**f**) RA signaling inhibition with the inverse agonist BMS493. In N2-stage embryos, *Pou3l* is expressed in the neural plate, except for the prospective cerebral vesicle, in the somites, and in the precursors of ectodermal sensory neurons located ventrally (arrows) (**a**,**c**,**e**). Expression is subsequently downregulated and is only found in the rhombospinal portion of the neural plate and the somites, most conspicuously in the first somite on the left side of the embryo (white arrows), at stage N4 (**b**,**d**,**f**). The treatments do not affect *Pou3l* expression. Whole mount embryos are shown in lateral (top) and dorsal (bottom) views; the anterior is to the left. The scale bar in (**a**) is 50 μm and is valid for all panels.

As previously reported [30], in N2-stage neurulae, *Pou3l* was expressed in almost the entire neural plate, excepting the prospective cerebral vesicle, in the somites, and in ventrally located precursors of ectodermal sensory neurons (Figure 3a, arrows). Later in development, at the N4 stage, expression was downregulated in most tissues and became limited to the rhombospinal portion of the nerve cord, with an inconspicuous signal remaining detectable in the somites, most conspicuously in the first left somite (Figure 3b, white arrow). We could not detect *Pou3l* expression at later stages of development. Furthermore, treatments with RA and BMS493 did not affect the expression patterns of the *Pou3l* gene (Figure 3).

**Figure 4:**
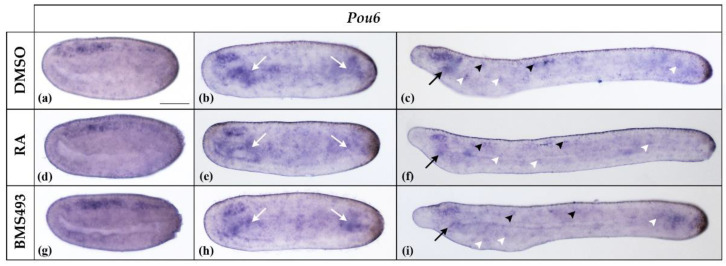
Expression of *Pou6* in N2-, N4-, and T1-stage *Branchiostoma lanceolatum* embryos. (**a**–**c**) Control embryos (DMSO-treated). (**d**–**f**) Retinoic acid (RA) signaling activation with all-*trans* retinoic acid (RA). (**g**–**i**) RA signaling inhibition with the inverse agonist BMS493. In N2 neurulae (**a**,**d**,**g**), *Pou6* is weakly expressed in the neural plate, with the strongest signal being located in its anterior half. At stage N4 (**b**,**c**,**h**), *Pou6* expression is detectable in the anterior nerve cord (in the region of the prospective cerebral vesicle) and in the endoderm (white arrows). In T1 embryos (**c**,**f**,**i**), *Pou6* is expressed in the cerebral vesicle, the anterior and posterior endoderm, including the preoral organ (black arrow), and in scattered cells of the nerve cord (black arrowheads) and the ectoderm (white arrowheads). Whole mount embryos are shown in lateral view; the anterior is to the left. The scale bar in (**a**) is 50 μm and is valid for all panels.

*Pou6* was expressed throughout the neural plate in N2-stage neurulae (Figure 4a). At stage N4, *Pou6* expression in the nerve cord was restricted to the anterior part, corresponding to the region of the prospective cerebral vesicle. In addition, *Pou6* was expressed throughout the endoderm, most conspicuously in the anterior and posterior parts (Figure 4b). At the T1 stage, *Pou6* transcription was maintained in the cerebral vesicle and scattered cells of the nerve cord as well as in the preoral organ and individual ectodermal cells (Figure 4c). A faint signal was also detectable in the anterior and posterior endoderm. RA signaling manipulation did not affect *Pou6* expression (Figure 4).

### 3.2. Pharmacological Disruption of Endogenous Retinoic Acid Signaling Differentially Affects Brn1/2/4 Expression in the Anterior Endoderm

**Figure 5:**
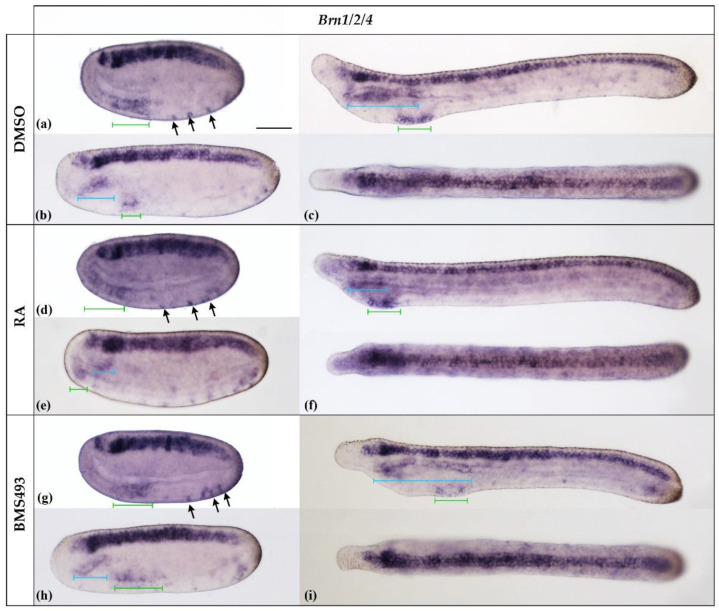
Expression of *Brn1/2/4* in N2-, N4-, and T1-stage *Branchiostoma lanceolatum* embryos. (**a**–**c**) Control embryos (DMSO-treated). (**d**–**f**) Retinoic acid (RA) signaling activation with all-*trans* retinoic acid (RA). (**g**–**i**) RA signaling inhibition with inverse agonist BMS493. *Brn1/2/4* is strongly expressed in the neural plate and ventral pharyngeal endoderm (green bars) as well as in differentiating ectodermal sensory neurons (arrows) at the N2 stage (**a**,**d**,**g**). This pattern is maintained at later stages, with the addition of a new expression domain in the dorsal anterior endoderm at the N4 stage (blue bars) (**b**,**e**,**h**), which persists at the T1 stage (**c**,**f**,**i**). Whole mount N2- and N4-stage embryos are shown in lateral views; T1-stage embryos are shown both in lateral (top) and dorsal (bottom) views. The anterior is to the left. The scale bar in (**a**) is 50 μm and is valid for all panels.

The amphioxus *Brn1/2/4* gene is homologous to the *Brn1*, *Brn2*, and *Brn4* genes of vertebrates [29]. In agreement with previous reports on *B. floridae*, the *B. lanceolatum Brn1/2/4* gene was expressed at the N2 stage in the entire neural plate, excepting a small gap in the center of the prospective cerebral vesicle, in developing ectodermal sensory neurons ventrally, and in the ventral part of the anterior archenteron (Figure 5a). By the N4 stage, *B. lanceolatum Brn1/2/4* expression extended dorsally in the anterior endoderm (Figure 5b) and became restricted, ventrally, to the territory of the prospective first pharyngeal gill slit (Figure 5b). At the T1 stage (Figure 5c), *B. lanceolatum Brn1/2/4* was strongly expressed in the nerve cord, from the posterior cerebral vesicle to the tail bud. A faint signal was also observed in the anterior tip of the cerebral vesicle. In the anterior endoderm, *B. lanceolatum Brn1/2/4* expression extended dorsally along most of the length of the pharynx, including the preoral organ, and remained restricted ventrally to the region of the prospective first gill slit.

Pharmacological perturbation of endogenous RA signaling levels did not alter the expression pattern of *B. lanceolatum Brn1/2/4* in the nerve cord (Figure 5). Conversely, the treatments impacted the development of the anterior endoderm: the pharynx was reduced following RA treatment and enlarged in the presence of the RAR inverse agonist BMS493, as previously described [44]. Following the treatments, expression of *Brn1/2/4* was maintained dorsally in the anterior part and ventrally in the posterior part of the anlagen of the pharynx. The size of the dorsal expression domain was reduced upon RA treatment and expanded in BMS493-treated embryos when compared to controls (Figure 5c,f,i). The position of the ventral domain was shifted anteriorly by RA and posteriorly by BMS493, and this effect was visible as early as the N2 stage (Figure 5a–i). The treatments also affected the anteroposterior extent of the ventral expression domain in neurula-stage embryos. At the T1 stage, however, the size of the expression domain was similar in all specimens (Figure 5a–i).

### 3.3. Interference with Endogenous Retinoic Acid Signaling Levels Differentially Affects Amphioxus Pou4 Expression along the Anteroposterior Axis of the Developing Nerve Cord

**Figure 6:**
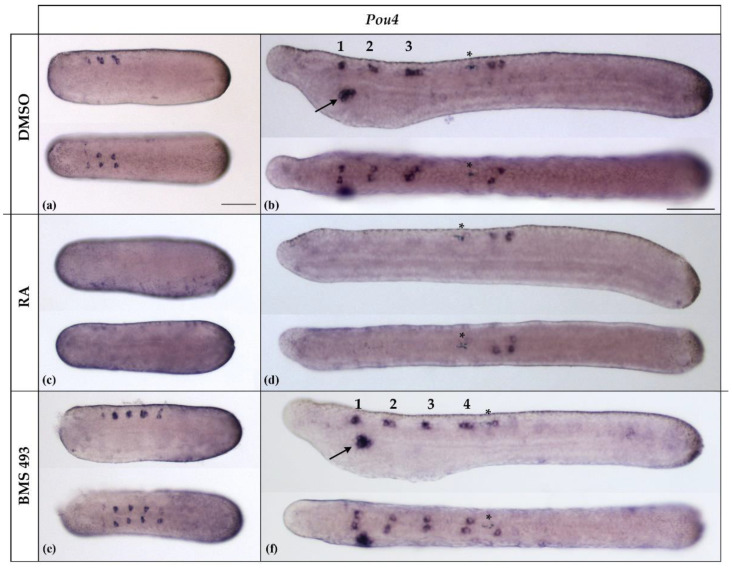
Expression of *Pou4* in N4- and T1-stage *Branchiostoma lanceolatum* embryos. (**a**,**b**) Control embryos (DMSO-treated). (**c**,**d**) Retinoic acid (RA) signaling activation with all-*trans* retinoic acid (RA). (**e**,**f**) RA signaling inhibition with the inverse agonist BMS493. In N4-stage neurulae, *Pou4* was expressed in three pairs of neurons in the anterior nerve cord and scattered ectodermal sensory neurons (**a**). At this stage, *Pou4* expression was completely lost in the nerve cord following RA treatment (**c**), while BMS493 treatments induced supernumerary pairs of *Pou4*-positive cells in the nerve cord (**e**). At the T1 stage (**b**,**d**,**f**), *Pou4* was further expressed in a variable number of neural cells posterior to the first pigment spot (asterisks) and in the mouth primordium (arrow). *Pou4* expression in the mouth primordium was lost in RA-treated embryos (**d**) and maintained in BMS493-treated embryos (**f**). Whole mount embryos are shown in lateral (top) and dorsal (bottom) views; the anterior is to the left. The scale bar in (**a**) is 50 μm and is valid for panels (**a**,**c**,**e**); the scale bar in (**b**) is 50 μm and is valid for panels (**b**,**d**,**f**).

**Figure 7:**
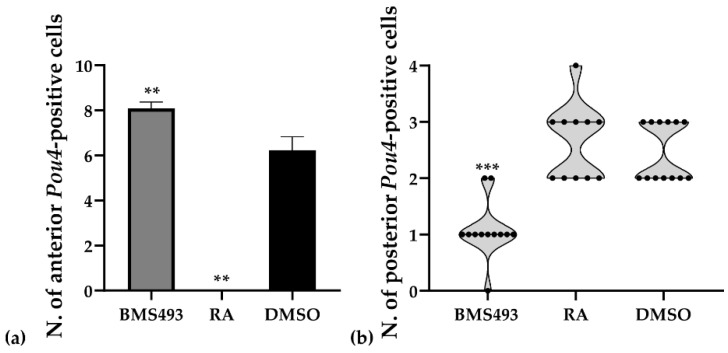
Effects of retinoic acid (RA) signaling manipulation on the number (N.) of cells expressing *Pou4* in the nerve cord anterior and posterior to the first pigment spot at the T1 stage. (**a**) The average number of *Pou4*-positive cells in the nerve cord anterior to the first pigment spot is indicated by bars and the standard deviation by error bars. (**b**) The number of *Pou4*-positive cells posterior to the first pigment spot is presented in the form of a violin plot with each dot representing a specimen. DMSO, treatment control (*n* = 13); BMS493, RA signaling inhibition with an inverse agonist (*n* = 12); RA, RA signaling activation with all-*trans* retinoic acid (*n* = 11); **, *p* < 0.01; ***, *p* < 0.001.

In *B. lanceolatum*, *Pou4* was initially expressed at the N4 stage in differentiating ectodermal sensory neurons and in three bilateral pairs of neurons located in the anterior half of the nerve cord (Figure 6a). These expression domains were largely maintained at the T1 stage, with the paired *Pou4*-positive cells occupying very specific anteroposterior positions in the anterior half of the nerve cord (Figure 6b). While the anterior *Pou4*-positive cells were located in the dien-mesencephalon, at the level of the caudal end of somite 1, the central and posterior *Pou4*-positive pairs were located in the rhombospinal region, respectively, at the level of somite 2 and somite 3. For the two *Pou4* expression sites in the rhombospinal region, at the N4 neurula stage, there was generally one pair of *Pou4*-positive cells per site (10/12, 83.33%), with one cell on each side of the hemicord. However, in rare cases, there were three (two in one and one in the other hemicord, 1/12, 8.33%) or four (two per hemicord, 1/12, 8.33%) *Pou4*-positive cells (Figure 6b and Figure 7a). At the T1 stage, *Pou4* expression became detectable in additional cells in the rhombospinal region, just posterior to the first pigment spot, at the level of the junction between somite 4 and somite 5. In addition to the signals in the nerve cord and ectodermal sensory cells, at the T1 stage, *Pou4* was further expressed in the primordium of the mouth (Figure 6b).

The expression of *Pou4* was significantly affected by treatments with RA and BMS493. Thus, in embryos treated with RA, the expression of *Pou4* in the nerve cord anterior to the first pigment spot was completely lost (11/11, 100%, *p* < 0.01), together with *Pou4* expression in the mouth primordium (Figure 6c,d). Conversely, embryos treated with the inverse agonist BMS493 were characterized by supernumerary *Pou4*-positive cells in the rhombospinal region of the nerve cord (12/12, 100%, *p* < 0.01), located anterior to the first pigment spot and thus anterior to the level of somite 5 (Figure 6e,f). Similar to what we observed in controls, some BMS493-treated embryos (1/12, 8.33%) featured more than one *Pou4*-positive cell per hemicord at a given anteroposterior position in the rhombospinal region (Figure 7a).

The number of *Pou4*-positive cells in the nerve cord located posterior to the first pigment spot at the T1 stage was variable between specimens, with control embryos featuring either two (7/13, 53.85%) or three (6/13, 46.15%) cells expressing *Pou4*. These cells were present on both sides of the midline, with a preference for the right hemicord in specimens with three cells (5/6, 83.33%).Treatment with RA predominantly produced embryos with either two or three cells expressing *Pou4*, with identical ratios for two or three cells (5/11, 45.45%). Only one RA-treated specimen was characterized by four cells (1/11, 9.09%). No statistically significant differences were thus observed between control animals and those treated with RA (*p* > 0.9). In contrast, the inhibition of RA signaling activity with BMS493 resulted in a clear reduction of the number of *Pou4*-positive cells in the nerve cord at levels posterior to the first pigment spot, with most specimens exhibiting only a single *Pou4*-positive cell (9/12, 75.00%, *p* < 0.001) (Figure 7b).

**Figure 8:**
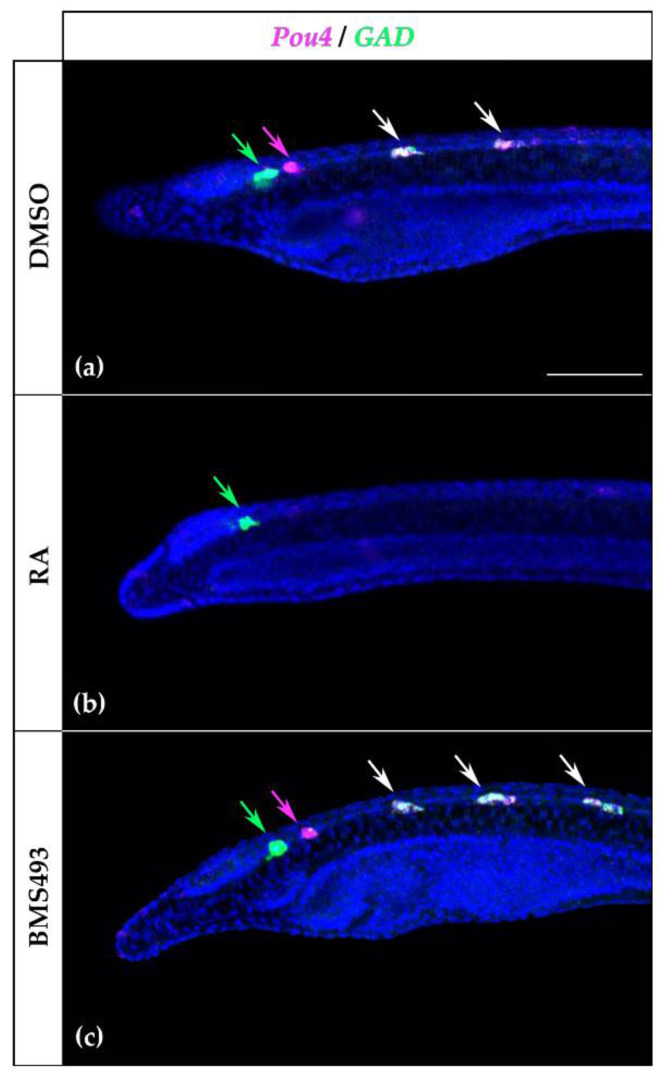
Co-localization analysis of *Pou4* and *GAD* in T1-stage *Branchiostoma lanceolatum* embryos. (**a**) Control embryos (DMSO-treated). (**b**) Retinoic acid (RA) signaling activation with all-*trans* retinoic acid (RA). (**c**) RA signaling inhibition with the inverse agonist BMS493. *Pou4*-expressing cells are in magenta and highlighted by magenta arrows, *GAD*-expressing cells are in green and highlighted by green arrows, and cells expressing both genes are in white and highlighted by white arrows. Cell nuclei are labeled with Hoechst and shown in blue. The anterior part of each embryo is presented in lateral view with the anterior to the left and the dorsal side up. For each embryo, a z-projection of the focal planes containing the nerve cord is shown. The scale bar in (**a**) is 50 μm and is valid for all panels.

Since the expression pattern of *Pou4* in the rhombospinal region of the nerve cord was reminiscent of that of *GAD*, which encodes the glutamate decarboxylase enzyme crucial for the synthesis of the neurotransmitter GABA [13], and since it has previously been described that *GAD* expression is controlled by RA signaling in amphioxus [21], we decided to investigate the possible co-expression of *Pou4* and *GAD* in the nerve cord by in situ HCR. At the T1 stage, the first pair of cells expressing *Pou4* was located posterior to the first pair of cells expressing *GAD* (Figure 8a), which were not affected by treatment with RA (Figure 8b). The second and third pairs, however, co-expressed *Pou4* and *GAD,* and treatment with RA completely suppressed the expression of both genes. Furthermore, the supernumerary *Pou4*-positive cells induced by BMS493 treatment also co-expressed *GAD* (Figure 8c).

## 4. Discussion

In this work, we performed pharmacological manipulations of RA signaling in developing amphioxus to investigate the regulation of POU genes by RA signaling. Our results indicate that RA signaling controls aspects of *Brn1/2/4* expression in the pharynx and of *Pou4* in the nervous system. Although POU genes are known to be important mediators of animal development, information regarding the conservation of their expression and function across metazoans remains fragmentary. Therefore, we will first discuss the developmental expression patterns of POU genes in amphioxus in a comparative and evolutionary context before discussing the effects of RA signaling on the expression of amphioxus *Brn1/2/4* and *Pou4*.

### 4.1. Evolutionary Considerations on the Expression of POU Genes in Metazoans

A *Pit-1* (*Pou1f1*) ortholog is present in the genomes of cnidarians, indicating an early metazoan origin of POU I genes, with apparent secondary losses of members of this class in ecdysozoans and multiple spiralian lineages [46]. In cnidarians, *Pit-1* is expressed in a subpopulation of differentiating sensory cells of the rhopalia in the jellyfish *Aurelia aurita*, suggesting that sensory cell development might have been an ancestral function of this gene in metazoans [21]. In vertebrates, *Pit-1* expression is strongly associated with the adenohypophysis [47,48]. Furthermore, transcriptome analyses in different murine cells and tissues have identified high levels of *Pit-1* expression in myeloid and erythroid cell lineages [49]. In amphioxus, *Pit-1* expression is limited to the preoral organ, the homolog of the vertebrate adenohypophysis [26,50], suggesting that expression in blood cells might be a vertebrate novelty. In rodents, *Pit-1* expression is regulated by an RA-dependent enhancer [51], and in chicken, the development of the adenohypophyseal placode (Rathke’s pouch) requires RA signaling [52]. In contrast, our data suggest that the expression of *Pit-1* in the developing preoral organ in amphioxus is not affected by RA signaling, although the region of the endoderm giving rise to the preoral organ requires low levels of RA for correct development [24].

Members of the POU II subfamily have so far only been identified in bilaterian genomes [1]. Vertebrates have three *Pou2* paralogs. *Pou2f1* (*Oct1*) is expressed in most cell types, while *Pou2f2* (*Oct2*) is strongly expressed in lymphoid cells and neuroectodermal lineages [49,53]. *Pou2f3* (*Oct11*) expression is limited to the ectoderm and acts in the differentiation of keratinocytes and taste bud sensory cells in mice [54,55]. We found that the only amphioxus POU II subfamily member, *Pou2*, is characterized by a highly dynamic developmental expression pattern, with an early phase of ubiquitous expression that becomes increasingly restricted as development proceeds. The ubiquitous expression of *Pou2* at the N2 stage thus resolves into tissue-specific expression in the endoderm, the prospective cerebral vesicle, and the tail bud at the N4 stage, and ultimately becomes limited to the pharyngeal endoderm at the T1 stage.

POU III genes are expressed in the nervous system in a variety of animal taxa, such as acoels, cnidarians, insects, and vertebrates [56], suggesting deeply conserved roles for POU III genes in the control of neural functions. Conserved expression across bilaterians is also found outside the nervous system, in particular in excretory organs [57,58]. During vertebrate development, all four *Pou3* paralogs are thus expressed in the nervous system. In frogs, *Pou3f4* (*Brn4*) is also expressed in the Spemann–Mangold organizer, and its misexpression causes an ectopic activation of neural genes [59]. In mammals, *Pou3f1* appears to be the key factor for neural fate promotion, while the only neural defect caused by the loss of *Pou3f4* is deafness [60]. This suggests that, although class III POU genes have conserved roles in neural specification and development, a certain degree of interchangeability between paralogs arose during vertebrate evolution. Amphioxus has two class III POU genes, which likely originated from a single ancestral chordate *Pou3* gene by a cephalochordate-specific duplication event [18]. In *B. lanceolatum*, the onset of *Pou3l* expression seems to be earlier than that of *Brn1/2/4*, with *Pou3l* transcripts being first detectable at the G2 stage and *Brn1/2/4* only becoming detectable at the N2 stage [30]. In *B. floridae*, however, *Brn1/2/4* expression was detected as early as the G2 stage [29], and *Brn1/2/4* rather than *Pou3l* was proposed to be an upstream factor in the putative neural gene regulatory network of G5-stage gastrulae [30]. Similar to the situation in other bilaterians, we further found that one of the two amphioxus POU III genes, *Pou3l*, was detectable in a developing excretory organ of amphioxus: *Pou3l* was transiently expressed in the somites at early neurula stages, and, as the somitic signal faded, *Pou3l* expression persisted in the first somite on the left side of the embryo, in a domain corresponding to the site of formation of Hatschek’s nephridium, the initial amphioxus kidney [61]. This expression of *Pou3l* in Hatschek’s nephridium is comparable to that of *Pou3* in the excretory organs of a wide variety of bilaterian animals and thus lends further support to the hypothesis that ultrafiltration-based excretory organs evolved only once, in the last common ancestor of bilaterians [58].

In several jawed vertebrates, *Brn1* (*Pou3f3*) is expressed in the mesenchyme of the anterior pharyngeal arches that derive from the neural crest [62]. Conversely, in the lamprey, a jawless vertebrate, the *Pou3c* gene is only diffusely expressed in the pharyngeal region at later development stages, but its expression is not enriched in the arch mesenchyme [62]. While the functions of *Pou3* genes in the pharynx have not yet been elucidated in the lamprey, in jawed vertebrates, *Brn1* has been implicated in the development of the tissue covering the gill arches, and co-option of its expression from the CNS to the pharyngeal mesenchyme has been proposed as the evolutionary event allowing the formation of a gill cover, which is present in jawed vertebrates and absent from lampreys [62]. In amphioxus, which lacks a definitive neural crest [9], *Brn1/2/4* is expressed from early neurula stages in the anlagen of the amphioxus pharyngeal gill slits, which are homologous to the pharyngeal arches of vertebrates. Although the pharyngeal gill slits of amphioxus are covered by tissue, this gill cover is probably not homologous to that of jawed vertebrates. Indeed, the gill cover of amphioxus originates from the ventral body wall located posterior to the pharynx [63], while those of cartilaginous and bony fishes are derived from the most anterior arches [62]. These data are consistent with the notion that, while *Pou3* expression in the pharynx is likely an ancestral chordate trait, its enrichment in arch mesenchyme and role in gill cover development evolved later, in an early jawed vertebrate [62].

Class IV POU genes are found in all metazoans excepting the ctenophores [1] and are predominantly expressed in the nervous system where they regulate the terminal differentiation of neurons, often of a sensory type. In the fruit fly *Drosophila melanogaster*, *Pou4*/*Acj6* regulates the function of olfactory neurons [64]. Similarly, *Unc-86*, the *Pou4* ortholog of the nematode *Caenorhabditis elegans*, is expressed by around one-fifth of the neurons of the CNS, and its mutation alters the animal’s response to odors [65,66,67]. In mammals, there are three *Pou4* genes (*Pou4f1*/*Brn3a*, *Pou4f2*/*Brn3b*, and *Pou4f3*/*Brn3c*), all of which are expressed in the nervous system, most prominently in specific sensory organs [68]. Knock-out experiments in mice revealed, for example, that *Pou4f2* is required for the development of mechanosensory hair cells in the auditory and vestibular systems [69,70] and that *Pou4f3* is necessary for the specification of retinal ganglion cells [70,71]. *Pou4* is also expressed in chemo- and mechanosensory neurons of embryos and larvae of both cephalochordates [31] and tunicates [72], and it is likely that at least the role of *Pou4* in terminal differentiation of mechanosensory neurons predates the origin of bilaterians [73].

*Pou5f1*, better known as *Oct3/4*, is a key regulator of pluripotency in mammals and one of the so-called Yamanaka factors [74,75]. Class V POU genes are only found in vertebrates and are thought to have originated from an ancient *Pou3*-like gene [1]. Interestingly, *Pou3* is expressed in circulating stem cells of tunicates, the sister group of vertebrates, suggesting that the newly originated vertebrate *Oct3/4* might have replaced *Pou3* in an ancestral pluripotency program that was in place before the split between tunicates and vertebrates [76]. Although little is known about stem cells in amphioxus, the most prominent stem cell niche so far identified during development is in the tail bud and comprises at least the primordial germ cells [77]. Excepting the transient expression of *Pou2* during neurulation, none of the amphioxus POU genes are expressed in the tail bud. Although further studies are needed, our data thus suggest that the involvement of *Pou3*/*Pou5* genes in pluripotency is not a chordate synapomorphy.

Vertebrates have two class VI POU genes. *Pou6f1* is expressed weakly in neuroectoderm and germ cells of developing mice and broadly in the CNS, heart, lung, liver, kidney, skin, immune cells, and sperm of adults [49]. Relative to that of *Pou6f1*, the expression of *Pou6f2* is weak and restricted to specific regions of the CNS [49,68]. In non-vertebrate bilaterians, expression of *Pou6* orthologs was only reported in the CNS of the pygmy squid [56]. In developing amphioxus, we found *Pou6* to be most prominently expressed in the endoderm and the nerve cord. It might thus be that class VI POU genes have an ancient role in the development of bilaterian nervous systems. However, additional work assessing the expression of *Pou6* genes in a much wider variety of animals will be required to further investigate this hypothesis.

### 4.2. Retinoic Acid-Dependent and -Independent Brn1/2/4 Expression in the Developing Pharynx

Our analyses revealed that, while *Brn1/2/4* expression in the developing amphioxus nerve cord was not regulated by RA signaling, its expression in the endoderm was affected by treatments with both RA and BMS493. This finding is consistent with the previously described role of RA signaling in establishing the posterior limit of the amphioxus pharynx, a process involving the regulation of *Hox1* activity in the foregut endoderm [23]. RA is synthesized in the posterior part of the embryo [78], and diffusion creates a gradient with RA concentrations decreasing from posterior to anterior. In addition, the anterior pharyngeal endoderm expresses *Cyp26-2*, which encodes an RA-degrading enzyme, whose activity is necessary for the correct patterning of this tissue [79]. Consequently, the endoderm is divided into an anterior domain, which corresponds to the pharynx and develops in a low-RA environment, and a posterior domain characterized by high levels of RA [24].

*Brn1/2/4* was expressed both ventrally and dorsally in the pharyngeal endoderm, with ventral expression being detectable starting at the N2 stage and dorsal expression at the N4 stage. While the effects of RA and BMS493 treatments on the dorsal domain of the pharyngeal endoderm were directly correlated with the anatomical phenotypes, with RA reducing and BMS 493 expanding the signals along the anteroposterior axis, the effects on the ventral domain were more complex. At the N2 stage, exogenous RA expanded and shifted anteriorly the ventral expression of *Brn1/2/4*, while treatment with BMS493 slightly reduced it. Later, in N4 neurulae, *Brn1/2/4* expression in the ventral endoderm was restricted to the posterior part of the prospective pharynx. The effects of RA signaling manipulation were more evident at this stage: the ventral *Brn1/2/4* domain was expanded posteriorly by treatments with BMS493 and shifted anteriorly by exogenous RA administration. In T1-stage embryos, *Brn1/2/4* was expressed ventrally at the level of the primordium of the first gill slit, marking the posterior end of the pharynx. The size of this expression domain of *Brn1/2/4* was unaffected by the treatments.

These data suggest that early expression of *Brn1/2/4* in the ventral endoderm might serve a role in specifying ventral endoderm, while later expression in the ventral endoderm might be involved in gill slit morphogenesis. In this scenario, the early changes in *Brn1/2/4* expression induced by RA signaling alterations in the ventral endoderm are directly correlated with *Hox1*-dependent regional patterning of the endoderm along its anteroposterior axis [23]. Conversely, the late expression of *Brn1/2/4* in the ventral endoderm, visible at the T1 stage, is unaffected by the treatments and restricted to the anlagen of the first gill slit, whose position changes in the pharyngeal endoderm of treated embryos due to the regional *Hox1*-dependent mispatterning that occurred earlier in development. Late *Brn1/2/4* expression in the ventral endoderm might thus function in gill slit formation, a process that has been proposed to require only low levels of endogenous RA signaling [24].

### 4.3. Evidence for Pou4 as a Terminal Selector of Neuron Types in Amphioxus

*Pou4* genes regulate the neurotransmitter identity of specific neuron populations in a variety of metazoans. In the fruit fly, for example, *Pou4*/*Acj6* defines cholinergic identity in olfactory sensory neurons by direct binding to the cholinergic gene locus, which encodes the acetylcholine-synthesizing enzyme ChAT (choline acetyltransferase) and the acetylcholine transporter VAChT (vesicular acetylcholine transporter) [80]. In the nematode worm *C. elegans*, *Pou4/Unc-86* directly regulates the expression of *Vacht*, *Vglut* (encoding the vesicular glutamate transporter), and *Tph* (encoding tryptophane 5-hydroxylase, the rate-limiting enzyme in serotonin synthesis) in cholinergic, glutamatergic, and serotoninergic neurons, respectively [81]. Similarly, in adult mice, *Brn3a*/*Pou4f1* is necessary for the maintenance of terminal identity in medial habenular neurons, including the expression of *Vglut1* in glutamatergic neurons and *Chat* in cholinergic neurons [82], and in ascidian tunicate larvae, *Pou4* is expressed in both dorsal and ventral peripheral neurons of the tail [72], which also express the *Tph* gene [83].

Of all amphioxus POU genes, *Pou4* had the most restricted expression pattern in the nervous system. *Pou4* was only expressed in a group of two to three cells located posterior to the first pigment spot and, more anteriorly, in three pairs of segmentally arranged neurons. Of these, the most anterior pair was in the dien-mesencephalon, while the other two pairs were in the rhombospinal region of the nerve cord. We showed that the neurons expressing *Pou4* in the rhombospinal region, but not those located in the dien-mesencephalon, co-expressed *GAD* and were therefore GABAergic. Given their position just posterior to the first pair of GABAergic neurons, the most anterior cells expressing *Pou4* likely correspond to the third large paired neurons (LPN3), which are glutamatergic [13,84]. In the PNS, only a subset of sensory neurons that expressed *Pou4* also expressed *GAD*, indicating that some of the *Pou4*-positive cells in the PNS are not GABAergic. It is likely that at least a part of these *Pou4*-positive/*GAD*-negative ectodermal sensory neurons, in particular those located in the mid-trunk region, are glutamatergic [22]. *Pou4* thus seems to be involved in the differentiation of at least four different neuron types in amphioxus: glutamatergic neurons in the dien-mesencephalon, GABAergic interneurons in the rhombencephalon-spinal cord, and glutamatergic and GABAergic sensory neurons in the PNS. Although gene knockout and misexpression experiments are required to functionally validate these results, it is likely that *Pou4* acts as a terminal selector for these neuronal subtypes in amphioxus, as is the case in fruit flies, nematodes, and mice [80,81,82].

Our treatment results indicate that RA signaling, either directly or indirectly, negatively regulates *Pou4*, which in turn promotes *GAD* expression and acquisition of GABAergic identity in the rhombospinal region. Regulation of *GAD* expression in the rhombospinal region of the amphioxus nerve cord by RA signaling has been shown to be correlated with RA-induced changes in *Hox3* expression [21]. RA signaling might thus be involved in setting the posterior limit of the region of the nerve cord competent to produce GABAergic neurons. Alternatively, based on the observation that RA can antagonize *Pou4f2* (*Brn3b*) expression in murine neuroblastoma cells [32], RA signaling might act directly on specific subsets of neural progenitor cells in the developing amphioxus CNS by repressing *Pou4* expression, hence blocking the activation of *GAD* transcription. Further experiments, including genetic manipulation of *Pou4*, are necessary to test whether *Pou4* directly controls *GAD* expression and to gain a deeper understanding of this process.

Our results further revealed a cryptic neuron diversity in the amphioxus CNS. Although segmentally arranged and evenly spaced, the three pairs of GABAergic neurons in the anterior nerve cord are specified by different genetic programs, suggesting that they might also have different functions. An in-depth investigation of the molecular composition of larval neurons will be required to better define neuron populations in the developing amphioxus nerve cord and to infer homologies with specific neuron types in the vertebrate CNS.

### 4.4. A Graded Response to Retinoic Acid Signaling Regionalizes the Developing Amphioxus Nerve Cord

**Figure 9:**
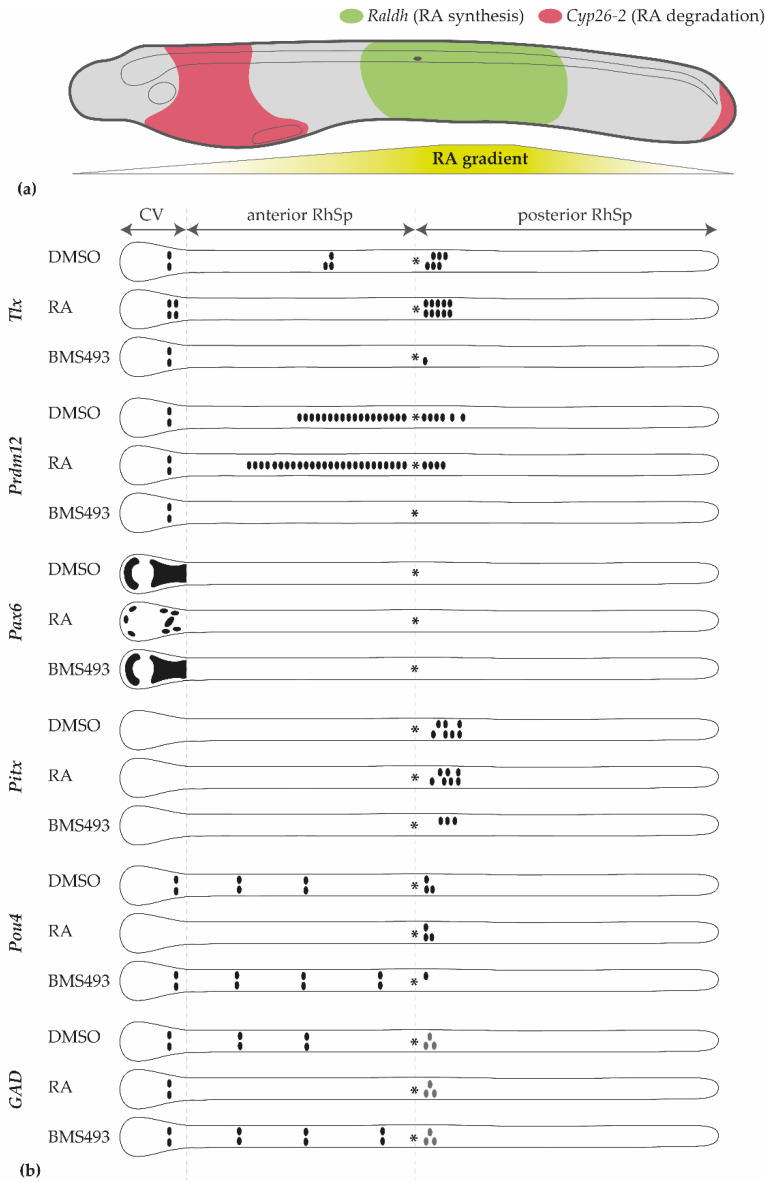
Model of retinoic acid (RA)-dependent regionalization of the amphioxus nerve cord. (**a**) Schematic illustration of the RA gradient in T1-stage embryos as deduced from *Raldh* and *Cyp26-2* expression. The embryo is shown in lateral view, with the anterior on the left. (**b**) Schematic representation of the expression patterns of *Tlx*, *Prdm12*, *Pax6*, *Pitx*, *Pou4*, and *GAD* in the central nervous system of T1-stage embryos in controls (DMSO) and following treatments to upregulate (RA) or inhibit (BMS493) RA signaling activity. Each outline represents the nerve cord of an embryo in dorsal view, with black shapes and dots indicating gene expression and asterisks marking the position of the first pigment spot. The *GAD* expression domain posterior to the first pigment spot is shown in gray to indicate that the number of cells varies at the T1 stage. CV: cerebral vesicle; RhSp: rhombospinal region. References for expression data: *Raldh* [78]; *Cyp26-2* [79]; *Tlx*, *Prdm12*, *Pax6*, and *Pitx* [45]; *GAD* [21].

Our data are consistent with the existence of a graded response to RA signaling in the amphioxus nerve cord. In vertebrate embryos, the opposing activities of RA-synthesizing and RA-degrading enzymes create an RA signaling gradient along the anteroposterior axis. A similar gradient is likely in place in developing amphioxus, where *Cyp26-2* is expressed in the anterior part of the embryo, and *Raldh* genes are expressed in the posterior half of the embryo (Figure 9a) [78,79]. In particular, three regions can be distinguished in the developing amphioxus nerve cord based on their responsiveness to RA signaling alterations (Figure 9b): the cerebral vesicle (homologous to parts of the vertebrate forebrain and midbrain [10]), the rhombospinal region anterior to the first pigment spot, and the rhombospinal region posterior to the first pigment spot. RA upregulation results in gene misexpression in the cerebral vesicle (*Pax6* and *Tlx* [45], *Pou4*) and in the anterior rhombospinal region (*Tlx* and *Prdm12* [45], *Pou4*) but does not affect the region posterior to the first pigment spot (*Pitx* and *Prdm12* [45], *Pou4*). Conversely, RA downregulation impacts gene expression in both the anterior (*Tlx* and *Prdm12* [45], *Pou4*) and posterior (*Pitx* and *Prdm12* [45], *Pou4*) rhombospinal regions but not in the cerebral vesicle (*Pax6*, *Tlx* and *Prdm12* [45], *Pou4*). These observations can be reconciled with a model in which neuronal specification requires very low levels of RA signaling in the cerebral vesicle, intermediate levels of RA signaling in the anterior rhombospinal region, and high levels of RA signaling in the posterior rhombospinal region.

However, it is noteworthy that the response to the alteration of RA signaling is not homogeneous within a given region. For example, treatment with RA is able to downregulate *Pax6* expression and completely suppress the expression of *Pou4* in the cerebral vesicle but has no effect on the expression of *Prdm12* and *GAD* and, remarkably, promotes the formation of an additional pair of *Tlx*-expressing cells [45]. Likewise, treatment with BMS493 produces more cells expressing *Pou4* but suppresses the expression of *Prdm12* and *Tlx* in the anterior rhombospinal region. Individual cell populations along the anteroposterior axis of the amphioxus nerve cord are thus characterized by cell-type-specific responses to the RA gradient. RA signaling therefore might not only have a role in the global regionalization of the nerve cord but also in specifying particular neural subtypes within the CNS [19].

## 5. Conclusions

POU genes are important regulators of cell type specification in animals. The control of their expression dynamics during development is therefore crucial for the correct differentiation of embryonic, larval, and adult structures. One signaling molecule that is capable of modulating the expression of specific POU genes in vitro is RA. Despite indirect evidence suggesting that RA signaling can also regulate POU genes transcription in vivo, studies investigating RA-dependent regulation of POU genes in developing embryos are lacking. This work is amongst the first to address this shortcoming by providing evidence that RA signaling regulates the expression of *Brn1/2/4* and *Pou4* in the endoderm and the nerve cord, respectively, of developing amphioxus. Since amphioxus represents the earliest-branching chordate, this information is also relevant for our understanding of the origin of the regulation of POU genes by RA. Given the paucity of studies investigating the relationship between RA signaling and POU genes, two hypotheses can be proposed: (1) RA-dependent regulation of POU genes, as seen in living amphioxus, arose early in chordate evolution or (2) RA-dependent regulation of POU genes is a cephalochordate apomorphy, not shared with other chordates. Based on the observation that RA can modulate the expression of specific POU genes in vertebrate cell cultures, the first hypothesis seems more likely. Future work investigating the roles of RA signaling during vertebrate development will be invaluable for resolving this question.

## Data Availability

Data are contained within the article and the Supplementary Materials.

## Supplementary Materials

The following supporting information can be downloaded at: https://www.mdpi.com/article/10.3390/cells12040614/s1. Table S1: Sequence of the primers used in this study. Figure S1: Control in situ hybridization experiments with sense probes for *Pou2* and *Pou6*.
