# Supplementary material for: Retinoic Acid and POU Genes in Developing Amphioxus: A Focus on Neural Development
Source: Cells. 2023 Feb 14;12(4):614. doi: 10.3390/cells12040614 (PMC9953854; doi:10.3390/cells12040614)
Supplement: Supplementary file 1 [file cells-12-00614-s001.zip › cells-2156080-supplementary.pdf]

Table S1. Sequence of the primers used in this study.

Pit-1 forward	5′-GTCCGCACCTGCCTTTCCCCTACC-3′
Pit-1 reverse	5′-GCCACGCGCACGATGTCCTGAGA-3′
Pou2 forward	5′-CAACGGGTCAGTGAATGGGATAGA-3′
Pou2 reverse	5′-CCGCTTGGTGGAGGGATGAC-3′
Pou3l forward	5′-TCACCGCCGCACCTGGAGAA-3′
Pou3l reverse	5′-TGCCGTGCCCGTTGGTCATC-3′
Brn1/2/4 forward	5′-GGTCTTCGCCCCTGCTTAGTAGTC-3′
Brn1/2/4 reverse	5′-TCGTTGTTTTGGCCCCTGGTAGTC-3′
Pou4 forward	5′-ATGCACCCTCCAATAACTGCCG-3′
Pou4 reverse	5′-TTATCTCCCGCCTTGCTGCG-3′
Pou6 forward	5′-TCTGAACGCCGTCCACTCTATGAA-3′
Pou6 reverse	5′-GTCTGGCGGGCGAAGCGTTTGTG-3′

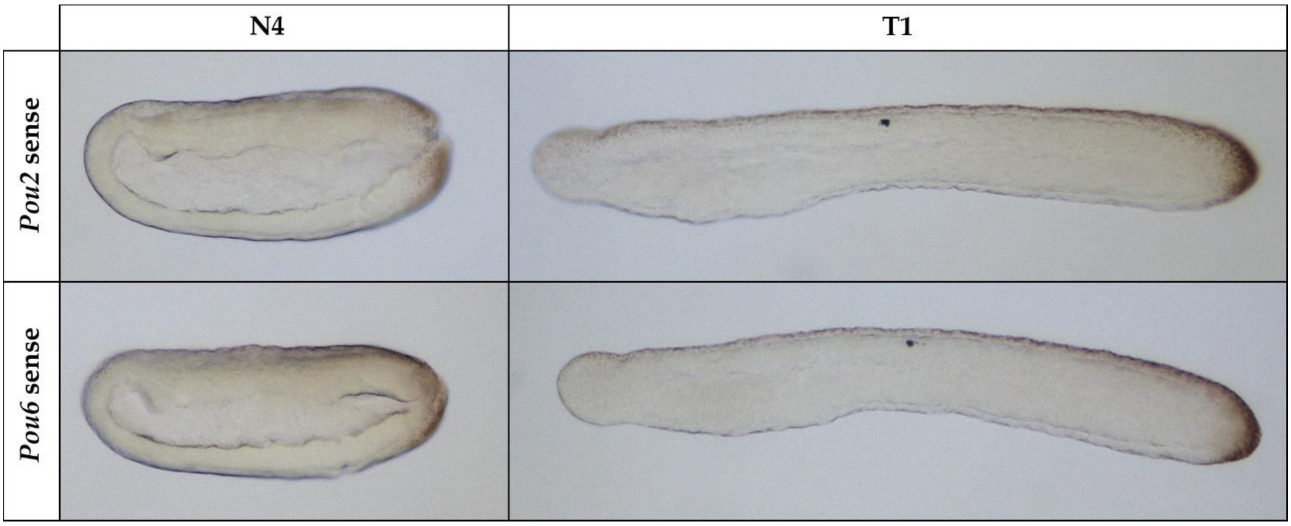

Figure S1. Negative control *in situ* hybridization with sense probes for *Pou2* and *Pou6*. No signal is detected in control embryos after the same experimental protocol that yielded a specific signal with the antisense probes shown in Figures 2 and 4.
